# Supplementary material for: Transcriptome Profile Reveals Differences between Remote and Ischemic Myocardium after Acute Myocardial Infarction in a Swine Model
Source: Biology (Basel). 2023 Feb 21;12(3):340. doi: 10.3390/biology12030340 (PMC10045039; doi:10.3390/biology12030340)
Supplement: Supplementary file 1 [file biology-12-00340-s001.zip › Supplementary Figure S1.pdf]

Supplementary Figure S1: Cardiac magnetic resonance study. Images show delayed enhancement four chamber views of a representative infarcted myocardium at day 3 post infarction (A) and the same animal imaged at the end of the study (10 days) (B). The infarcted area is indicated by white arrows, mainly in the anteroseptal myocaridum. Main function cardiac parameters calculated where measures at these times and represented in the table. LVEF left ventricular ejection fraction, EDVi end diastolic volume indexed to body surface area, ESVi end systolic volume indexed to body surface area. Infarct area is expressed as % of the left ventricle(C).

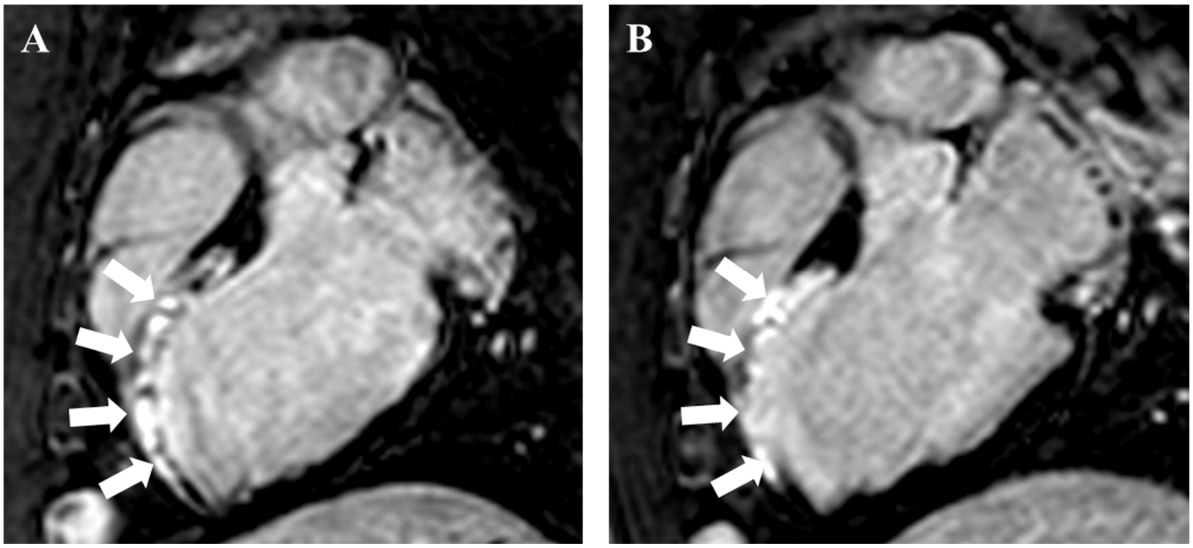

C

	LVEF (%)		%MI		EDVi (ml/m^2)		ESVi (ml/m^2)	
**Animal**	**3 days**	**10 days**	**3 days**	**10 days**	**3 days**	**10 days**	**3 days**	**10 days**
**#1**	26.00	31.00	22.00	20.00	96.50	116.57	71.37	80.91
**#2**	27.00	30.00	21.00	20.00	80.48	96.81	59.00	68.02
**#3**	23.00	37.00	23.00	20.00	117.26	99.74	90.61	62.95
**#4**	21.50	23.31	14.00	13.00	87.19	73.55	68.45	56.40
**Mean**	24.37	30.33	20.00	18.25	95.36	96.67	72.36	67.07
